# Transcriptome Analysis and SNP Development Can Resolve Population Differentiation of *Streblospio benedicti*, a Developmentally Dimorphic Marine Annelid

**DOI:** 10.1371/journal.pone.0031613

**Published:** 2012-02-16

**Authors:** Christina Zakas, Nancy Schult, Damhnait McHugh, Kenneth L. Jones, John P. Wares

**Affiliations:** 1 Department of Genetics, University of Georgia, Athens, Georgia, United States of America; 2 Department of Biology, Colgate University, Hamilton, New York, United States of America; 3 Department of Biochemistry and Molecular Genetics, University of Colorado School of Medicine, Aurora, Colorado, United States of America; Emory University School of Medicine, United States of America

## Abstract

Next-generation sequencing technology is now frequently being used to develop genomic tools for non-model organisms, which are generally important for advancing studies of evolutionary ecology. One such species, the marine annelid *Streblospio benedicti*, is an ideal system to study the evolutionary consequences of larval life history mode because the species displays a rare offspring dimorphism termed *poecilogony*, where females can produce either many small offspring or a few large ones. To further develop *S. benedicti* as a model system for studies of life history evolution, we apply 454 sequencing to characterize the transcriptome for embryos, larvae, and juveniles of this species, for which no genomic resources are currently available. Here we performed a *de novo* alignment of 336,715 reads generated by a quarter GS-FLX (Roche 454) run, which produced 7,222 contigs. We developed a novel approach for evaluating the site frequency spectrum across the transcriptome to identify potential signatures of selection. We also developed 84 novel single nucleotide polymorphism (SNP) markers for this species that are used to distinguish coastal populations of *S. benedicti*. We validated the SNPs by genotyping individuals of different developmental modes using the BeadXPress Golden Gate assay (Illumina). This allowed us to evaluate markers that may be associated with life-history mode.

## Introduction

Investigating trade-offs in life history of marine taxa has greatly informed our understanding of macroevolutionary outcomes such as taxonomic diversification, geographic range size and rate of extinction ([1], [2]; reviewed in [3], [4]). Understanding the molecular and regulatory mechanisms that underlie these trade-offs can make a considerable contribution to our understanding of life history evolution. This is particularly true for marine invertebrates, where variation in life history traits such as offspring size and number is orders of magnitude greater than that for terrestrial animals [5]. Different species of marine invertebrates often exhibit alternative reproductive strategies that either maximize adult fecundity or offspring survival [3], [5], [6]. *Planktotrophic* species produce large numbers of tiny eggs where larvae feed in the plankton for weeks to months, potentially traveling 100 s of kilometers, resulting in extended ranges and high gene flow [7]–[12]. *Lecithotrophic* taxa produce fewer, larger eggs that are maternally endowed with enough energy to complete development; their non-feeding larvae have reduced dispersal, but higher survival. By influencing gene flow, larval type has sweeping micro- and macroevolutionary impacts on a lineage [13], [14]. Larval type can influence potential for local adaptation [15], population genetic structure [16]–[20], rate of protein evolution [21], and evolutionary processes such as speciation and extinction ([1], [16] reviewed in [22]); they are also tied to global patterns of marine diversity [23]. Here we are developing a remarkable model system for studying how a dimorphic life history strategy is maintained within a single species.

The common estuarine polychaete *Streblospio benedicti* is a particularly interesting species for exploring the evolution of contrasting life histories because there are two distinct yet heritable larval types that can occur together in coastal populations along the US East Coast [24]–[26]. With a developmental polymorphism known as *poecilogony* [27], females of *S. benedicti* can produce either hundreds of small eggs that develop into planktotrophic feeding larvae with a long development time (∼150 eggs of 60 µm diameter), or tens of large eggs that develop lecithotrophically, maturing quickly while feeding on maternally provided yolk (∼40 eggs of 100 µm diameter) (in lab studies, these large-egg larvae have been observed taking up food particles at a later developmental stage [28]). In addition to the contrast in their initial size, the two larval forms differ in the formation of larval bristles [29], and in the timing of gut development [24], [28]. Ultimately, the different larvae develop into indistinguishable juveniles. Thus *S. benedicti* provides a rare and largely unexplored opportunity to study a suite of developmental trade-offs, including larval size, larval duration, and maternal investment within a single species where potentially confounding interspecies comparisons are minimized.

To develop *S. benedicti* as a model system for life history comparisons at the genetic level, we use the transcriptome of a pooled set of embryos, larvae and juveniles from large-egg lecithotrophic females from San Pedro, CA (SP). We have two main goals for this transcriptome analysis: (1) We address the utility of applying molecular population genetic indices to whole transcriptome data and assess the match of these inferences to known characteristics of the source population. Specifically, for a non-normalized transcriptome, we have the opportunity to investigate the ability of summary statistics based on the site frequency spectrum (SFS) to reflect the relative influences of evolutionary mechanisms such as historical demography and selection (reviewed in [30]). Analyzing the SFS allows us to detect signatures of selection in regions of the transcriptome that may affect larval mode and differentiate locally adapted populations. (2) In addition, we develop informative single nucleotide polymorphism (SNP) markers for further differentiation between geographic and phenotypic populations of *S. benedicti* for subsequent studies.

Given the paucity of genomic data for marine polychaetes (there are few annelid datasets in NCBI's genbank: 1.4×10^4^ sequence submissions and 515 popsets for the Annelida compared to 1.4×10^6^ protein and 1.2×10^4^ popsets for the Arthropoda as of Feb 2011), we are as yet unable to annotate this transcriptome to the degree of other model systems; the genome for *Capitella teleta* has just been released (among the first for the Lophotrochozoa) but *C. teleta* is more than 400 million years divergent from *S. benedicti* [31]. We therefore focused primarily on polymorphism diversity within a single reduced-complexity genomic sample. This allowed us to establish early benchmarks for regions of the genome that potentially harbor the additive and expression variance underlying the observed life-history variation.

## Methods

### Transcriptome Generation

Large-egg *Streblospio benedicti* adults were collected by Dr. Bruno Pernet from the intertidal zone near San Pedro, CA and maintained in Petri dishes in artificial sea water with a fine layer of mud at 22–23°C, and with a 12-hour day/night cycle as described by [32]. No specific permits were required for the described field studies, as the location is not privately-owned or protected and *S. benedicti* is not an endangered or protected species. All embryos and larvae were dissected from the maternal dorsal brood pouches using an Olympus SZ61 dissecting microscope.

Total RNA from 325 embryo, larvae and juvenile individuals that were collected across many developmental time points was isolated using an RNAqueous-Micro Kit (Ambion), and ds-cDNA was prepared according to the SMARTer Pico PCR cDNA Synthesis Kit (Clontech). The cDNA library was not normalized prior to sequencing, and sequence data were generated using a quarter plate GS titanium chemistry FLX Next-gen (Roche 454) by the University of York Technology Facility.

Sequence read files were aligned *de novo* in SeqMan sequence assembler (DNASTAR) using the default alignment settings (minimum match percent = 80, minimum sequence length = 100 bp, match size = 12). We determined this set of parameters was appropriate based on preliminary analyses with a range of parameter sets (minimum match percent = 85, 75) that did not greatly change the number of quality contigs generated (results not shown).

### Characterization of Assembly

To identify potential signatures of selection across individual assembled contigs, we calculated summary statistics based on the SFS, which is the distribution of nucleotide frequencies at a large number of loci. We tested for contigs that represented outlier SFS patterns by calculating Tajima's D (D_T_) [33] and Fu and Li's F* [34] for each contig using the program Compute [35]. Assuming some major violations to the standard test (e.g. elevated sequencing error, etc.) we choose to focus more on the outliers of this distribution rather than the deviation from the null expectation itself [36], [37]. The best BLAST hit and the predicted gene ontology (GO) terms (GO; The Gene Ontology Consortium 2000) were determined for each of these contigs using Blast2GO [38] with a GO *e* value of 1.0 e^−6^ [39]. Using the GO information we determined what category of gene functions were represented in the highly positive and negative D_T_ and F* categories.

To analyze the overall SFS of our transcriptome, we compared the distribution of actual minor allele frequencies (MAF) for identified SNPs to the predicted MAF distribution for a population under neutrality. To do this, we initially found all SNPs from the transcriptome using PipeMeta [40] with the minimum SNP site depth set to one, and a minimum nucleotide depth at a SNP site set to ten (n = 745). This initially liberal SNP criterion was applied to eliminate SNPs that are at such low frequency they are likely to be the product of sequencing error, while still including real low-frequency SNPs in the SFS analysis. These initially broad criteria insured that a large number of SNPs were included in our analysis, despite the likelihood that many of these singleton SNPs are actually the product of sequencing error. Because of the high sequence error rate associated with 454 sequencing (∼0.5%, [41]), we only chose nucleotide replacement SNPs and not insertion-deletion mutations. This is consistent for the entirety of the study. To determine the expected MAF under neutrality, we used the program **ms** [42] to simulate the same number of genealogies of sample size 10 that were restricted to a single segregating site (as with our SNPs). We then calculated π (pairwise differences between sequences) for each replicate and estimated the MAF for each replicate. We compared the expected and observed MAF distribution for the two data sets using a chi-squared test.

### SNP Marker Development

We choose SNP markers that will be informative for future population genetic studies based on *a priori* criteria. To design the most informative markers, we wanted to balance our choice of SNPs between highly conserved housekeeping genes and genes that are more typically associated with ecological and physiological function. For the latter category we were specifically interested in SNPs that occur in gene regions known to exhibit high protein diversity in natural populations (*e.g.*, allozyme loci listed in [43] and genes involved in gut development [44], [45].

Choosing SNPs in regions with good BLAST scores may bias our selection towards genes that are highly conserved across a diverse set of organisms and thus less variable in general. To mitigate ascertainment bias, we chose to balance the proportion of SNPs with good BLASTn hits (*e*-value of less than 10^−10^) against the NCBI nucleotide database, and others with less informative BLAST hits, which may be due to omission of annelid genes from NCBI's database, or significant sequence divergence from taxa currently represented in GenBank. SNPs with low MAFs are less likely to be represented in the transcriptome, and therefore choosing SNPs based on the MAF in the SP sample will introduce ascertainment bias when applied to other populations [46]. We therefore choose SNPs with a range of MAFs to equally represent four categories of MAFs: 30–35%, 36–40%, 41–45% and 46–50%.

In addition, we chose to minimize the number of SNPs in the assay that are associated with ribosomal regions. From the BLAST results we were able to determine which SNPs were known to occur in ribosomal regions, although there is a small possibility that there may be additional ribosomal SNPs chosen that we were unable to identify.

We used BLASTn to determine whether the nucleotide substitution resulted in synonymous or non-synonymous changes in the protein sequence. We used NCBI's open reading frame (ORF) finder (www.ncbi.nlm.nih.gov/projects/gorf/) to determine all potential ORFs for each contig that contained one of the known SNPs. We determined the correct ORF by selecting the longest possible ORF and performing a BLASTx search on the resulting protein coding sequence and verified that it matched the initial nucleotide BLASTn result. When this was possible, and the SNP occurred within an open reading frame, the SNP was putatively scored as either a synonymous or non-synonymous substitution.

### SNP Discovery

The aligned contigs were used with the PipeMeta software package [40] using the default settings (as opposed to the settings described above for SFS analysis), as subsequent stringency criteria would later be applied. We wanted to ensure that the SNPs we chose for population genetic analyses were not the products of sequencing error, so we applied more strict criteria than in our initial SFS analyses. Here, our criteria for choosing SNPs require that the MAF was greater than 30%, and the coverage greater than 10×. Otherwise, we were not confident that the SNP was real [47]. We wanted to design SNPs for use with the Illumina Golden Gate assay, which allows high-throughput multiplex genotyping of SNPs. Therefore we also limited our selection to SNPs that had high probability of success with the Golden Gate technology. The SNPs were scored according to primer rankings generated by Illumina based on the 60 nucleotides flanking each side of the SNP. We also attempted to minimize potentially confounding effects of linkage disequilibrium by choosing only one SNP per contig for the final assay, although many of the SNP-containing contigs had multiple polymorphic loci. We used Arlequin 3.5 [48] to test for nonrandom associations between loci at a significance level of 5% level in both populations.

### Genotyping

Individuals were collected from both SP, and the Baruch Institute of Marine Biology, SC (BR) and genotyped using the BeadXPress Golden Gate assay (Illumina) to verify the 96 SNPs. All 46 of the SP individuals are lecithotrophic and of the 50 BR individuals 17 are definitive lecithotrophs (which were observed releasing lecithotrophic larvae) and 33 are putative lecithotrophs (which appeared to be brooding lecithotrophic larvae that were not ready for release). These individuals were used to verify the 96 SNPs. Whole specimen genomic DNA was isolated as in [49]. Nucleic acid quality and concentration were evaluated with a Nanodrop ND-1000 spectrophotometer. Golden Gate genotyping (Illumina) was conducted on 50 ng of DNA according to manufacture's protocols at the Georgia Genomics Facility (dna.uga.edu).

Genotypes were assigned and annotated using GenomeStudio (Illumina) with a default SNP call threshold of 0.30 (on a scale of 0–1). The call threshold is based on the distance of an individual read from the center of the SNP call cluster. We also evaluated a more stringent call threshold of 0.45, and while this significantly reduced the successful call rate for each SNP, it did not change the results of subsequent population analyses (data not shown). For the two populations genotyped and at all loci, we calculated observed and expected heterozygosity, as well as standard F-statistics, using Arlequin 3.5 [48]. Significance of statistics was assayed through standard permutation tests of 10,000 iterations.

## Results

The 454 pyrosequencing generated 336,715 reads of ∼400 bp average length. The SeqMan alignment produced 7,222 total contigs with an average contig length of 436 base pairs and 3.08× coverage. Singleton reads were excluded. PipeMeta found 2,817 SNPs (2,095 were biallelic SNPs) in total from the 7,222 contigs, although 6,940 contigs contained no nucleotide substitution SNPs. SNPs that failed the criterion of having no more than two possible nucleotides were excluded from our analysis.

### Transcriptome Analysis

**Figure 1:**
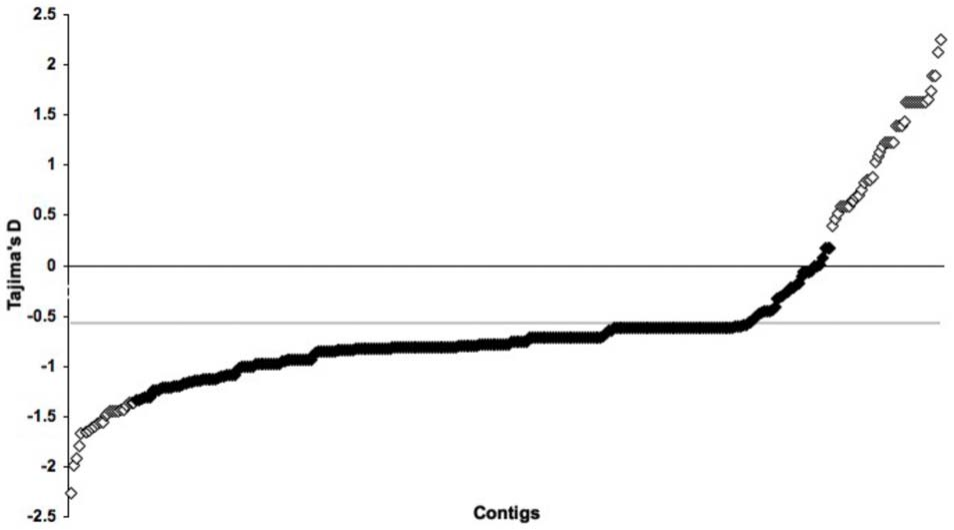
D_T_ distributions for all contigs. The grey line is the mean D_T_. White diamonds are contigs with D_T_ values greater or less then the standard deviation from the mean.

Of the contigs that produced D_T_ values (*i.e.* had sufficient polymorphism and coverage), no D_T_ statistics were significant relative to a null coalescent simulation model. The average D_T_ for the transcriptome was −0.574 with a SD of 0.774, as opposed to previously analyzed mtCOI data from East and West Cost populations that had a D_T_ of −1.98 [50], [51]. There were 42 contigs that were greater than one standard deviation above the average and 25 that were more negative than one standard deviation below (Figure 1). Within the ‘biological process’ designation of GO terms, under the most inclusive category (level 2), the majority of sequences were implicated in metabolic and cellular processes. No apparent difference in the representation of categories of GO terms was observed between the high and low D_T_ contigs (data not shown). We found no difference in F* except that 71 more contigs occurred in the positive tail of the distribution. The average F* across the data set was 0.51 (SD 0.92) and there was still no apparent difference in the distribution of GO terms for contigs with excessively positive and negative F*.

**Figure 2:**
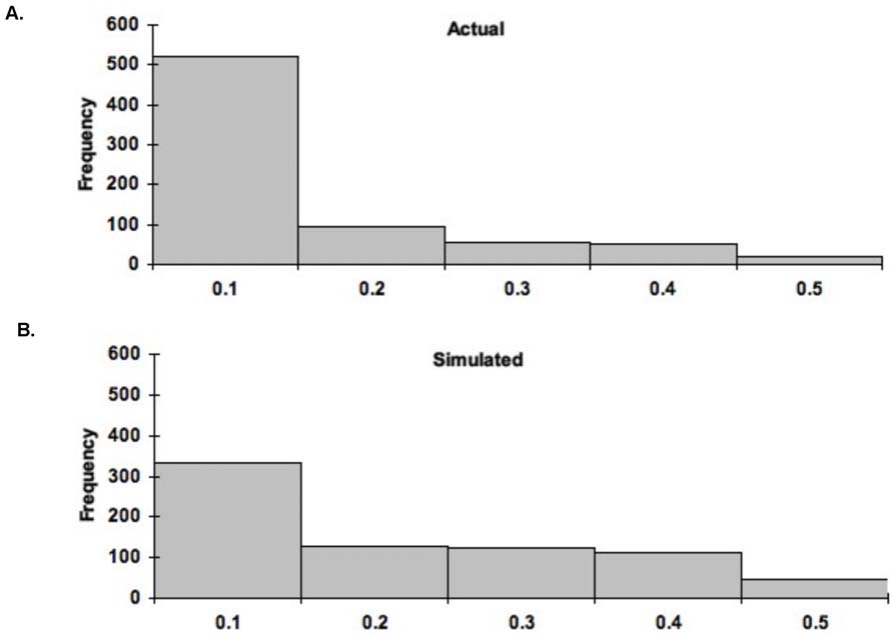
Histogram of the SFS for (A) actual and (B) simulated MAFs. Distributions are significantly different (*p*<<0.001).

When the observed transcriptome and simulated MAFs were compared in order to analyze the SFS we found a significant difference (chi-squared; p<0.01) between the observed and neutrally simulated values (Figure 2). There is an excess of rare alleles in the transcriptome SFS. Again, the excess number of single SNP substitutions may be more due to sequencing error than real allele frequencies and could lead to overestimating the apparent importance of purifying or directional selection [47]. Because this may skew our results we removed the MAF = 10% category and repeated the statistical comparison; the two distributions were still significantly different (p<0.01). This difference between the actual transcriptome SFS and that simulated under neutrality suggests that the population sampled in the transcriptome may not be evolving neutrally. However, it is difficult to determine the cause of this shift from neutral expectations as both the excess of low-frequency alleles and the negative D_T_ may be consistent with purifying or variable selection (see [37] and refs therein) or population expansion since introduction.

### SNP Categories

Application of our minimum depth and frequency criterion further reduced the number of candidate SNPs to 685. Finally, the Illumina primer quality scores designated 266 SNPs with an optimal rank (1) for successful primer design. Genes of functional interest, including developmental gut genes and heterozygous allozyme loci, were not represented in any of the contigs with a high probability (*e*-value of less than 10^−5^). Because none of the contigs had significant D_T_ values, and there was no difference in the functional categories assigned to genes that were in either tail of the D_T_ distribution, we could not confidently assign contigs to putatively neutral or selective classes. Because we wanted to equally represent each of our MAF categories within the framework of our selection criteria, we selected 19 SNPs with a MAF between 30–35%, 24 between 36–40%, 31 between 41–45% and 22 between 46–50%.

There were 37 SNPs that had a strong BLAST score (*e*-value less than 10^−10^; Table S1). All of these SNPs blasted to metazoans with 16 of the top hits belonging to animals in the Lophotrochozoa. We choose an additional 59 SNPs whose entire contig had a BLAST *e*-value of greater than 10^−10^. Of the 37 SNPs with strong BLAST scores, we chose nine that were ribosomal and 28 that were associated with other genes. Therefore only 24% of the chosen SNPs with known genomic association are believed to be ribosomal. Of the 37 SNPs with good BLAST scores, 24 occurred in an open reading frame that could be identified. Twelve of these produced non-synonymous substitutions in the amino acid.

### Genotyping

**Figure 3:**
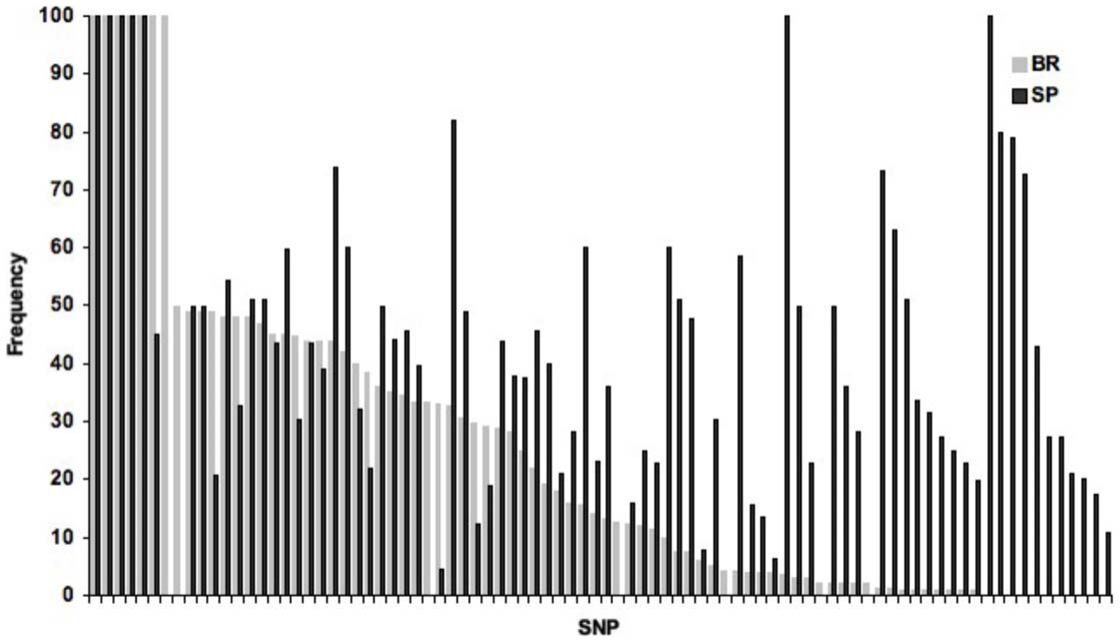
MAF distribution for each SNP at BR and SP.

Of the 96 SNPs originally chosen, ten of them (10.4%) produced no genotype calls in the assay and two were monomorphic and removed from subsequent analyses. The remaining 84 SNPs were validated [NCBI SRA SRA048717.1]. The average successful call rate for the remaining 84 SNPs was 87% (sd. 25%) of the individuals sampled, where 63 of the SNPs had call rates over 90% and an additional eleven were over 50% (Table S1). Three additional SNPs were monomorphic in both populations; however, these SNPs may be polymorphic in other populations. 16 SNPs were monomorphic in BR, and 12 SNPs were monomorphic in SP. There were 17 SNPs where the minor allele in BR was the major allele in SP (Figure 3). F_ST_ is 0.217 (p = 0.001) suggesting strong differentiation between the two populations. The F_IT_ for all markers is 0.197 (p<0.001), which also indicates population differentiation, although the F_IS_ (inbreeding coefficient within a population) is not significant. Significant nonrandom associations between loci pairs differed between the two populations. Of the 3570 pairwise comparisons between the 84 loci, in BR 1169 comparisons (32%) showed significant linkage disequilibrium, while in SP, only 184 loci (∼5%) were in significant linkage disequilibrium.

## Discussion

### Transcriptome Analyses

Our transcriptome analyses allow us to make preliminary identification of regions of the genome that potentially contain the sequence and expression variance that may underlie life-history variation. We used the SFS, which reflects the relative influences of evolutionary mechanisms such as historical demography and selection [52], to gain basic evolutionary insights for one population of *S. benedicti*. Because our transcriptome was not normalized, the resultant frequency of site variants is an unbiased estimate of true allele frequencies in the total population, assuming that expression is not dependent on allelic identity. Our EST library was generated from one population, SP, and we therefore expected the SFS to reflect a relatively neutral D_T_ ([53], [54] but see [55]), although it is important to note that SP was a recently introduced population (∼100ya) and may not represent a population at demographic equilibrium.

When generating the SFS we chose to use contigs with 10× coverage, as opposed to a higher coverage, to maximize cross-genome sampling. In this case it includes 745 total SNPs that meet our criteria. We included F* because it is more powerful in detecting the effect of background selection, as it is based on the difference between singleton mutations and the average number of nucleotide differences [34], but this result did not provide distinct insights from D_T_. However the SFS and D_T_ both indicate population expansion or purifying selection could be affecting the population; this signature is a common deviation from neutral expectations in metazoan taxa [37].

Our analyses of the SFS of lecithotrophic SP individuals show an overabundance of low frequency polymorphisms when compared to our neutral expectation (Figure 2), which may be consistent with population expansion or purifying selection. However, it is not clear that this is a true excess of rare alleles that is not simply due to sequencing error and sampling bias in a transcriptome of this coverage. More informative population inferences can be determined directly from the SNP analyses.

**Figure 4:**
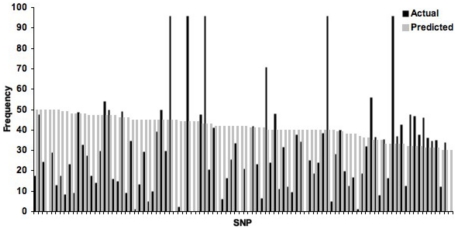
MAF distribution for each SNP in SP. Predicted values are calculated from the transcriptome MAF data and the actual MAF is from the population genotyping data.

It is clear that the MAF for a SNP in the transcriptome is not predictive of the true MAF in the population (Figure 4). This is not unexpected as the MAF from the transcriptome data is a product of randomly amplified reads and perhaps, with greater coverage, the actual MAF in the population would be better represented by the transcriptome. Additionally, there are a maximum of 46 individuals from SP that have genotype data for any given SNP, whereas in the transcriptome, there can be more variable coverage at a site, which may affect the MAF distribution. We may also expect true differentiation in MAF differences between small and large-egg populations. Through transcription profiling in *S. benedicti*, Marsh and Fielman [56] used a reannealing assay to demonstrate that small-egg individuals had a greater transcriptomic complexity with more inter-individual variation than large-egg individuals. This is consistent with findings that planktotrophic species in many taxa harbor greater diversity and lower dN/dS ratios than lecithotrophic species [57]–[59]. This suggests that the BR population, which harbors both larval types, could have a very different MAF for each SNP then the purely large-egg SP population. Marsh and Fielman [56] also predict that it is unlikely that two distinct developmental programs are harbored within this species, but rather small regulation differences in a few genes are likely responsible for the shift from planktotrophic to lecithotrophic individuals. This is the case in sea urchins, where the switch from planktotrophy to lecithoptrophy includes transcriptional changes that result in differences in cleavage patterns, axis specification, morphogenesis, and gene expression [60]–[64]. A similar regulation pattern may be occurring between the two *S. benedicti* larval types. These predictions remain to be tested in *S. benedicti*, but the SNPs developed here will allow us to compare allelic diversity across larval types.

### SNP development

From the transcriptome of *S. benedicti* we successfully developed 84 SNPs for use in population genetic and demographic studies. Because our cDNA library was not normalized prior to 454 pyrosequencing, there is an overabundance of ribosomal and other housekeeping genes. We wanted to ensure the SNPs chosen were representative of other transcribed genes as well. We were particularly interested in using SNPs that would be useful in differentiating coastal populations in future studies. Because the SP population that our transcriptome was generated from is a recently introduced population, the SNPs that occur are likely to be a subset of those in the source populations. To the extent possible, we chose SNPs that are representative of the entire transcriptome, rather than only the high copy number and housekeeping genes. Although we sampled only a single SNP from each assembled contig, there are still instances of significant linkage disequilibrium among some of our loci. The number of significant comparisons between SNP loci differed between our two populations by 27%, suggesting that distinct demographic or selective forces may be associated with the observed linkage disequilibrium rather than merely physical associations. There is not a significant inbreeding coefficient in these populations, although the recent introduction of the SP population, and the potential for a recent bottleneck, may be affecting our detection of linkage disequilibrium. Interestingly, of the SNPs that had significant BLAST results to the same gene region (Table S1) the only pair that showed significant linkage disequilibrium are SNPs 63 and 11 (actin genes) in BR.

There are 32 SNPs that were not in HWE in one or both populations. While this could be due to a variety of evolutionary mechanisms at these markers, it is possible that these SNPs have been shifted from expected frequencies due to genotyping call error. When we increased the stringency of the call rate in Illumina's GenomeStudio from 0.30 (default) to 0.45, we found that many of these SNPs (55%) lost genotyping calls completely and 21% of the SNPs did not change their expected heterozygosity at all. Of the remaining SNPs whose expected heterozygosity did change slightly, the difference between observed and expected heterozygosity remained significantly different. This demonstrates that increasing the call rate threshold may remove some SNPs with poor calls, but it does not affect the fixation indices (F_ST_, F_IS_, F_IT_). Therefore the expected fit to HWE will not change with increasing call stringency (data not shown). Interestingly, the proportion of genotyped individuals is not a good proxy for determining which SNPs will drop genotyping calls with increasing stringency. Instead, SNPs with a high expected heterozygosity (He>0.35) may be products of poor genotype assignments and generally drop out of the analysis when call stringency is increased.

It is important to note the transcriptome was generated from one lecithotrophic population (SP), while the SNP analysis used lecithotrophic individuals from two populations (SP and BR). Therefore the differences we see from this initial SNP analysis may only be due to population differences and not larval-type differences. It is notable that most SNPs have very different allele frequencies in both populations, although both populations are predominately the large-egg type (Figure 3). It is possible that some of the SNPs that are not informative in differentiating populations in this initial analysis will become important for differentiating larval types, and exploration of larval-type by population differentiation is the focus of ongoing studies.

The evidence for genetic structure between the two populations suggests one of two possibilities. These populations may have become more evolutionarily divergent since their separation ∼100ya. However it is unlikely that significant divergence between the populations occurred in such a short time. It is far more likely that the West Coast introduction(s) occurred from a population on the East Coast that harbored existing genetic differences from the one studied. It is possible that a few individuals established West Coast populations, or that SP has undergone a bottleneck since introduction. This suggests that there may be more genetic structure on the US East Coast than previously suggested [50] especially if the West Coast introduction originated from a population that has not previously been sampled, such as the Chesapeake Bay or further north.

Using SFS statistics, it seems that a transcriptome with this amount of coverage does not allow for definitive inferences about population demographics without greater coverage or more accurate knowledge of sequencing errors. More informative inferences can be drawn from the SNP markers themselves. The majority of these SNP markers have a high genotyping call rate and will be useful in differentiating genetic structure between geographic and phenotypic populations of *S. benedicti*. The SNP markers developed here will significantly improve our ability to investigate life history trade-offs in this species.

## Supporting Information

Table S1 **Categories and statistics for 84 SNPs.** SNPs that did not meet the criterion for BLAST *e-* values are left blank. For substitution type, Syn is a putatively synonymous substitution and NS is a putatively nonsynonymous substitution. – designates when an allele is fixed. SNPs that have a H_e_ (heterozygosity) over 0.35 generally dropped out all genotyping scores when the call stringency was increased. * is *p*<0.05, ** is *p*<0.01, *** is *p*<0.001.
(DOC)

## References

[1] Jablonski DJ (1986). Larval ecology and macroevolution in marine invertebrates.. Bull Mar Sci.

[2] Jablonski DJ, Lutz RA (1983). Larval ecology of marine benthic invertebrates: Paleobiological implications.. Biol Rev.

[3] Strathmann R (1985). Feeding and nonfeeding larval development and life history evolution in marine invertebrates.. Ann Rev Ecol Sys.

[4] Wray GA, McEdward LR (1995). Evolution of larvae and developmental modes.. Ecology of marine invertebrate larvae.

[5] Strathmann R (1990). Why life histories evolve differently in the sea.. Am Zoo.

[6] Strathmann R (1978). The evolution and loss of feeding larval stages in marine invertebrates.. Evolution.

[7] Levin LA, Bridges TS, McEdward LR (1995). Pattern and diversity in reproduction and development.. Ecology of marine invertebrate larvae.

[8] Caley M, Carr M, Hixon M, Hughes T, Jones G (1996). Recruitment and the local dynamics of open marine populations.. Ann Rev Ecol Sys.

[9] Todd CD (1998). Larval supply and recruitment of benthic invertebrates: Do larvae always disperse as much as we believe?. Hydrobiologia.

[10] Bohonak A (1999). Dispersal, gene flow, and population structure.. Quart Rev Biol.

[11] Pechenik J (1999). On the advantages and disadvantages of larval stages in benthic invertebrate life cycles.. Mar Ecol Prog Ser.

[12] Grosberg RK, Cunningham CW, Bertness MD, Gaines S, Hays ME (2001). Genetic structure in the sea: from populations to communities.. Marine community ecology.

[13] Hart M (2000). Phylogenetic analyses of mode of larval development.. Semin Cell Dev Biol.

[14] McEdward L (2000). Adaptive evolution of larvae and life cycles.. Semin Cell Dev Biol.

[15] Sotka E (2005). Local adaptation in host use among marine invertebrates.. Ecol Lett.

[16] Palumbi S (1994). Genetic divergence, reproductive isolation, and marine speciation.. Ann Rev Ecol Syst.

[17] Palumbi S, McEdward LR (1995). Using genetics as an indirect estimator of larval dispersal.. Ecology of marine invertebrate larvae.

[18] Hellberg M (1996). Dependence of gene flow on geographic distance in two solitary corals with different larval dispersal capabilities.. Evolution.

[19] Todd CD, Lambert J, Thorpe J (1998). The genetic structure of intertidal populations of two species of nudibranch molluscs with planktotrophic and pelagic lecithotrophic larval stages: Are pelagic larvae “for” dispersal?. J Exp Mar Biol Ecol.

[20] Collin R (2001). The effects of mode of development on phylogeography and population structure of North American *Crepidula* (Gastropoda: Calyptraeidae).. Mol Ecol.

[21] Foltz DW, Hrincevich AW, Rocha-Olivares A (2004). Apparent selection intensity for the cytochrome oxidase subunit I gene varies with mode of reproduction in echinoderms.. Genetica.

[22] Krug PJ (2011). Patterns of speciation in marine gastropods: A review of the phylogenetic evidence for localized radiations in the sea.. Am Malacol Bull.

[23] Thorson G (1950). Reproductive and larval ecology of marine bottom invertebrates.. Biol Rev Camb Philos Soc.

[24] Levin LA (1984). Multiple patterns of development in *Streblospio benedicti* Webster (Spionidae) from three coasts of North America.. Biol Bull.

[25] Levin LA, Huggett DV (1990). Implications of alternative developmental reproductive modes for seasonality and demography in an estuarine polychaete.. Ecology.

[26] Levin LA, Zhu J, Creed E (1991). The genetic basis of life-history characters in a polychaete exhibiting planktotrophy and lecithotrophy.. Evolution.

[27] Giard A (1905). La poecilogony..

[28] Pernet B, McHugh D (2010). Evolutionary changes in the timing of gut morphogenesis in larvae of the marine annelid *Streblospio benedicti*.. Evol Dev.

[29] Gibson G, MacDonald K, Dufton M (2010). Morphogenesis and phenotypic divergence in two developmental morphs of *Streblospio benedicti* (Annelid, Spionidae).. Invet Biol.

[30] Nielsen R (2005). Molecular signatures of natural selection.. Ann Rev Genetics.

[31] Peterson KJ, Cotton JA, Gehling JG, Pisani D (2008). The Ediacaran emergence of bilaterians: congruence between the genetic and the geological fossil records.. Phil Trans R Soc Lond B.

[32] Pernet B, McArthur L (2006). Feeding by larvae of two different developmental modes in *Streblospio benedicti* (Polychaeta: Spionidae).. Mar Biol.

[33] Tajima F (1989). Statistical methods for testing the neutral mutation hypothesis by DNA polymorphism.. Genetics.

[34] Fu YX, Li WH (1993). Statistical tests of neutrality of mutations.. Genetics.

[35] Thornton K (2003). libsequence: a C++ class library for evolutionary genetic analysis.. Bioinformatics.

[36] Luikart G, England PR, Tallmon D, Jordan S, Taberlet P (2003). The power and promise of population genomics: From genotyping to genome typing.. Nat Rev Gen.

[37] Wares JP (2010). Natural distributions of mitochondrial sequence diversity supports new null hypothesis.. Evolution.

[38] Conesa A, Götz S, García-Gómez JM, Terol J, Talón M (2005). Blast2GO: A universal tool for annotation, visualization and analysis in functional genomics research.. Bioinformatics.

[39] Barreto FS, Moy GW, Burton RS (2010). Intrapopulation patterns of divergence and selection across the transcriptome of the copepod *Tigriopus californicus*.. Mol Ecol.

[40] Vera JC, Wheat CW, Fescemyer HW, Frilander MJ, Crawford DL (2008). Rapid transcriptome characterization for a nonmodel organism using 454 pyrosequencing.. Mol Ecol.

[41] Margulies M, Egholm M, Altman WE, Attiya S, Bader JS (2005). Genome sequencing in microfabricated high-density picolitre reactors.. Nature.

[42] Hudson RR (2002). Generating samples under a Wright-Fisher neutral model.. Bioinformatics.

[43] Skibinski DOF, Ward RD (2004). Average allozyme heterozygosity in vertebrates correlates with Ka/Ks measured in human-mouse lineages.. Mol Biol Evol.

[44] Boyle MJ, Seaver EC (2008). Developmental expression of foxA and gata genes during gut formation in the polychaete annelid Capitella Sp I.. Evol Dev.

[45] Frobius AC, Seaver EC (2006). ParaHox gene expression in the polychaete annelid Capitella sp I.. Dev Genes Evol.

[46] Helyar SJ, Hemmer-Hansen J, Bekkevold D, Taylor MI, Ogden R (2011). Application of SNPs for population genetics of non-model organisms: new opportunities and challenges.. Mol Ecol Res.

[47] Lynch M (2009). Estimation of allele frequencies from high-coverage genome-sequencing projects.. Genetics.

[48] Excoffier L, Lischer HEL (2010). Arlequin suite ver 3.5: a new series of programs to perform population genetics analyses under Linux and Windows.. Mol Ecol Res.

[49] Doyle JJ, Doyle JL (1987). A rapid DNA isolation procedure for small quantities of fresh leaf tissue.. Phytochem Bull.

[50] Schulze SR, Rice SA, Simon JL, Karl SA (2000). Evolution of poecilogony and the biogeography of North American populations of the polychaete *Streblospio*.. Evolution.

[51] Mahon AR, Mahon HK, Dauer DM, Halanych KM (2009). Discrete genetic boundaries of three Streblospio (Spionidae, Annelida) species and the status of *S. shrubsolii*.. Mar Biol Res.

[52] Wakeley J (2008). Conditional gene genealogies under strong purifying selection.. Mol Biol Evol.

[53] Pannell JR (2003). Coalescence in a metapopulation with recurrent local extinction and recolonization.. Evolution.

[54] Ingvarsson PK (2005). Nucleotide polymorphism and linkage disequilibrium within and among natural populations of European aspen (*Populus tremula* L., Salicaceae).. Genetics.

[55] Städler T, Haubold B, Merino C, Stephan W, Pfaffelhuber P (2009). The impact of sampling schemes on the site frequency spectrum in nonequilibrium subdivided populations.. Genetics.

[56] Marsh AG, Fielman KT (2005). Transcriptome profiling of individual larvae of two different developmental modes in the poecilogonous polychaete *Streblospio benedicti* (Spionidae).. J Exp Zool Part B: Mol Dev Evol.

[57] Foltz DW (2003). Invertebrate species with nonpelagic larvae have elevated levels of nonsynonymous substitutions and reduced nucleotide diversity.. J Mol Evol.

[58] Foltz DW, Hrincevich AW, Rocha-Olivares A (2004). Apparent selection intensity for the cytochrome oxidase subunit I gene varies with mode of reproduction in echinoderms.. Genetica.

[59] Foltz DW, Mah CL (2010). Differences in larval type explains patterns of nonsynonymous substitutions in two ancient paralogs of the histone H3 gene in sea stars.. Evol Devol.

[60] Wray GA, Raff RA (1989). Evolutionary modification of cell lineage in the direct developing sea urchin *Heliocidaris erythrogramma*.. Dev Biol.

[61] Henry JJ, Raff RA (1990). Evolutionary change in the process of dorsoventral axis determination in the direct-developing sea urchin *Heliocidaris erythrogramma*.. Dev Biol.

[62] Raff RA, Sly BJ (2000). Modularity and dissociation in the evolution of gene expression territories in development.. Evol Dev.

[63] Emlet RB (1995). Larval spicules, cilia, and symmetry as remnants of indirect development in the direct developing sea urchin *Heliocidaris erythrogramma*.. Dev Biol.

[64] Haag EH, Sly BJ, Raff RA (1999). Apextrin, a novel extracellular protein involved in adaptive evolution of larval ectoderm direct-developing sea urchin *Heliocidaris erythrogramma*.. Dev Biol.

