# Supplementary material for: Transcriptome Analysis and SNP Development Can Resolve Population Differentiation of Streblospio benedicti, a Developmentally Dimorphic Marine Annelid
Source: PLoS One. 2012 Feb 16;7(2):e31613. doi: 10.1371/journal.pone.0031613 (PMC3281091; doi:10.1371/journal.pone.0031613)
Supplement: Table S1 — Categories and statistics for 84 SNPs. SNPs that did not meet the criterion for BLAST e- values are left blank. For substitution type, Syn is a putatively synonymous substitution and NS is a putatively nonsynonymous substitution. – designates when an allele is fixed. SNPs that have a He (heterozygosity) over 0.35 generally dropped out all genotyping scores when the call stringency was increased. * is p<0.05, ** is p<0.01, *** is p<0.001. (DOC) [file pone.0031613.s001.doc]

| SNP Name | SNP | BLAST (> *e* -10) | Sub Type | MAF BR | MAF SP | Predicted MAF | Proportion Genotyped individuals | BR Ho | BR He | SP Ho | SP He |
| --- | --- | --- | --- | --- | --- | --- | --- | --- | --- | --- | --- |
| 1 | T/C | ribosomal protein S9 | NS | 0.29 | 0.19 | 0.48 | 0.49 | 0.577 | 0.411* | 0.381 | 0.308 |
| 2 | A/C |  |  | 0 | 0.43 | 0.38 | 0.86 | - | - | 0.324 | 0.491* |
| 3 | T/A | 60s | NS | 0.438 | 0.738 | 0.35 | 0.98 | 0.542 | 0.492 | 0.261 | 0.340 |
| 4 | G/A | actin | Syn | 0.18 | 0.21 | 0.36 | 0.97 | 0.367 | 0.300 | 0.432 | 0.339 |
| 5 | T/A | mitochondrion |  | 0 | 0.79 | 0.35 | 1.00 | - | - | 0.022 | 0.328*** |
| 6 | T/A |  |  | 0.12 | 0.25 | 0.49 | 1.00 | 0.160 | 0.211 | 0.413 | 0.375 |
| 7 | C/A | alpha enolase gene | Syn | 0.14 | 0.23 | 0.5 | 0.94 | 0.204 | 0.245 | 0.415 | 0.356* |
| 8 | G/A |  |  | 0.4 | 0.32 | 0.45 | 0.92 | 0.233 | 0.478*** | 0.600 | 0.437* |
| 9 | A/G |  |  | 1 | 0.45 | 0.4 | 1.00 | - | - | 0.457 | 0.494 |
| 10 | G/A |  |  | 0.01 | 0.511 | 0.4 | 1.00 | 0.020 | 0.020 | 0.457 | 0.500 |
| 11 | G/A | actin | Syn | 0.021 | 0 | 0.45 | 0.94 | 0.043 | 0.042 | 0.000 | 0.000 |
| 12 | G/A |  |  | 0.042 | 0 | 0.44 | 0.98 | 0.083 | 0.080 | 0.000 | 0.000 |
| 13 | C/T |  |  | 0 | 0.2 | 0.48 | 1.00 | - | - | 0.022 | 0.328*** |
| 14 | C/G |  |  | 0.036 | 1 | 0.4 | 0.23 | 0.071 | 0.069 | 0.000 | 0.000 |
| 15 | C/A |  |  | 0.01 | 0.315 | 0.46 | 1.00 | 0.020 | 0.020 | 0.413 | 0.432 |
| 16 | G/A |  |  | 0.36 | 0.5 | 0.42 | 1.00 | 0.440 | 0.461 | 0.348 | 0.500* |
| 17 | A/G | Platynereis dumerilii EST |  | 0 | 1 | 0.31 | 0.03 | - | - | 0.000 | 0.000 |
| 18 | G/A |  |  | 0.01 | 0.227 | 0.4 | 0.96 | 0.021 | 0.021 | 0.318 | 0.351 |
| 19 | T/C | Tubulin beta-2C chain |  | 0.02 | 0 | 0.37 | 1.00 | 0.040 | 0.039 | 0.000 | 0.000 |
| 20 | G/T | ribosomal protein S11 |  | 0.074 | 0.511 | 0.47 | 0.97 | 0.064 | 0.138*** | 0.457 | 0.500 |
| 21 | G/C |  |  | 0.282 | 0.378 | 0.4 | 0.83 | 0.359 | 0.405 | 0.561 | 0.470 |
| 22 | A/G | Adenosylhomocysteinase B | NS | 0.122 | 0.159 | 0.45 | 0.94 | 0.245 | 0.215 | 0.317 | 0.267 |
| 23 | C/T |  |  | 0.02 | 0.359 | 0.47 | 1.00 | 0.040 | 0.039 | 0.500 | 0.460 |
| 24 | G/C |  |  | 0.439 | 0.435 | 0.41 | 0.99 | 0.429 | 0.493 | 0.391 | 0.491 |
| 25 | C/T | 60S ribosomal protein |  | 0.42 | 0.6 | 0.48 | 1.00 | 0.440 | 0.487 | 0.435 | 0.476 |
| 26 | T/C |  |  | 0.193 | 0.4 | 0.39 | 0.90 | 0.341 | 0.312 | 0.619 | 0.482 |
| 27 | G/A | beta tubulin | Syn | 0.383 | 0.22 | 0.45 | 0.57 | 0.767 | 0.473*** | 0.440 | 0.343 |
| 28 | C/T | heat shock protein 83 | Syn | 0 | 0.272 | 0.32 | 0.98 | - | - | 0.413 | 0.396 |
| 29 | A/G |  |  | 0 | 0.272 | 0.38 | 0.98 | - | - | 0.500 | 0.488 |
| 30 | C/A |  |  | 0.039 | 0.063 | 0.45 | 0.81 | 0.079 | 0.076 | 0.125 | 0.117 |
| 31 | T/C |  |  | 0.125 | 0 | 0.42 | 0.04 | 0.250 | 0.219 | 0.000 | 0.000 |
| 32 | T/A |  |  | 0.156 | 0.6 | 0.33 | 0.98 | 0.229 | 0.264 | 0.391 | 0.476 |
| 33 | C/T |  |  | 0.1 | 0.6 | 0.48 | 0.99 | 0.163 | 0.183 | 0.565 | 0.476 |
| 34 | T/G |  |  | 0.133 | 0.359 | 0.41 | 0.99 | 0.265 | 0.230 | 0.543 | 0.460 |
| 35 | C/T |  |  | 0.03 | 0.228 | 0.4 | 1.00 | 0.060 | 0.058 | 0.413 | 0.352 |
| 36 | C/T |  |  | 0.02 | 0.283 | 0.47 | 1.00 | 0.040 | 0.039 | 0.565 | 0.405** |
| 37 | T/C |  |  | 0 | 0.211 | 0.45 | 0.97 | - | - | 0.200 | 0.333** |
| 38 | C/T |  |  | 0.01 | 0.25 | 0.3 | 1.00 | 0.020 | 0.020 | 0.370 | 0.375 |
| 39 | C/T |  |  | 0.49 | 0.5 | 0.32 | 1.00 | 0.980 | 0.500*** | 1.000 | 0.500*** |
| 40 | C/A |  |  | 0.04 | 0.136 | 0.49 | 1.00 | 0.080 | 0.077 | 0.239 | 0.273 |
| 41 | T/G | Efi-alpha | NS | 0.011 | 0.63 | 0.47 | 0.97 | 0.021 | 0.021 | 0.478 | 0.466 |
| 42 | G/A | CaM mRNA for calmodulin | Syn | 0.49 | 0.207 | 0.3 | 0.99 | 0.939 | 0.500*** | 0.370 | 0.328 |
| 43 | T/C |  |  | 0.48 | 0.326 | 0.45 | 0.99 | 0.510 | 0.499 | 0.478 | 0.440 |
| 44 | G/A | ATP synthase alpha | NS | 0.449 | 0.598 | 0.45 | 0.99 | 0.490 | 0.495 | 0.500 | 0.481 |
| 45 | G/A |  | Syn | 0.01 | 0.198 | 0.4 | 0.97 | 0.020 | 0.020 | 0.209 | 0.317* |
| 46 | A/C |  |  | 0.25 | 0.375 | 0.45 | 0.96 | 0.500 | 0.375* | 0.659 | 0.469** |
| 47 | G/A |  |  | 0.115 | 0.228 | 0.33 | 0.98 | 0.146 | 0.203 | 0.326 | 0.352 |
| 48 | G/A | heat shock protein 70 (hsp70-3) | NS | 0.05 | 0.304 | 0.42 | 1.00 | 0.060 | 0.095** | 0.478 | 0.423 |
| 49 | C/A | ribosomal protein 60s | NS | 0.479 | 0.511 | 0.43 | 0.97 | 0.417 | 0.499 | 0.489 | 0.500 |
| 50 | C/(T) |  |  | 1 | 1 | 0.43 | 0.99 | - | - | 0.000 | 0.000 |
| 51 | T/A |  |  | 0.306 | 0.489 | 0.4 | 0.99 | 0.408 | 0.425 | 0.457 | 0.500 |
| 52 | T/C |  |  | 0.469 | 0.511 | 0.32 | 0.99 | 0.531 | 0.498 | 0.500 | 0.500 |
| 53 | T/G | Platynereis dumerilii EST transcription factor |  | 0.013 | 0.732 | 0.4 | 0.84 | 0.025 | 0.025 | 0.146 | 0.393*** |
| 54 | C/T |  |  | 0.48 | 0.543 | 0.46 | 1.00 | 0.960 | 0.499*** | 0.913 | 0.496*** |
| 55 | T/A |  |  | 0.074 | 0.477 | 0.42 | 0.95 | 0.106 | 0.138 | 0.409 | 0.499 |
| 56 | C/T |  |  | 0.333 | 0 | 0.38 | 0.49 | - | 0.444 | 0.000 | 0.000 |
| 57 | T/C | ATP synthase beta | Syn | 0.061 | 0.077 | 0.41 | 0.48 | 0.121 | 0.114 | 0.154 | 0.142 |
| 58 | A/G |  |  | 0.49 | 0.5 | 0.5 | 0.96 | 0.571 | 0.500 | 0.535 | 0.500 |
| 59 | C/A |  |  | 0.03 | 0.5 | 0.5 | 1.00 | 0.060 | 0.058 | 0.348 | 0.500* |
| 60 | A/G |  |  | 0.327 | 0.819 | 0.47 | 0.89 | 0.531 | 0.440 | 0.083 | 0.296*** |
| 61 | G/A | COI |  | 0 | 0.728 | 0.42 | 1.00 | - | - | 0.022 | 0.396*** |
| 62 | A/C |  |  | 0.5 | 0 | 0.4 | 0.02 | - | 0.500 | 0.000 | 0.000 |
| 63 | G/A | Actin mRNA | Syn | 0.446 | 0.303 | 0.31 | 0.88 | 0.587 | 0.494 | 0.605 | 0.422** |
| 64 | T/C | COI |  | 0.04 | 0.156 | 0.46 | 0.99 | - | 0.077*** | 0.000 | 0.263*** |
| 65 | A/(T) | 18s |  | 1 | 0 | 0.47 | 0.01 | - | - | 0.000 | 0.000 |
| 66 | G/(C) | 18s |  | 1 | 1 | 0.44 | 1.00 | - | - | 0.000 | 0.000 |
| 67 | A/C |  |  | 0.219 | 0.457 | 0.36 | 0.74 | 0.229 | 0.342* | 0.565 | 0.496 |
| 68 | C/T | beta-tubulin | Syn | 0.329 | 0.045 | 0.4 | 0.89 | 0.659 | 0.442** | 0.091 | 0.087 |
| 69 | G/A |  |  | 0.02 | 0.5 | 0.4 | 1.00 | 0.040 | 0.039 | 0.435 | 0.500 |
| 70 | G/A | beta-tubulin | Syn | 0.288 | 0.438 | 0.31 | 0.76 | 0.576 | 0.410* | 0.875 | 0.492*** |
| 71 | G/(A) | beta-tubulin | NS | 1 | 1 | 0.33 | 0.93 | - | - | 0.000 | 0.000 |
| 72 | T/C | mitochondrial RNA |  | 0 | 0.8 | 0.33 | 0.99 | - | - | 0.000 | 0.320*** |
| 73 | C/A |  |  | 0.041 | 0.587 | 0.5 | 0.99 | 0.082 | 0.078 | 0.565 | 0.485 |
| 74 | C/A |  |  | 0.01 | 0.272 | 0.5 | 0.99 | 0.020 | 0.020 | 0.457 | 0.396 |
| 75 | A/G | guanine nucleotide binding protein | NS | 0.35 | 0.442 | 0.32 | 0.97 | 0.340 | 0.455 | 0.279 | 0.493*** |
| 76 | T/C |  |  | 0.16 | 0.283 | 0.42 | 1.00 | 0.240 | 0.269 | 0.478 | 0.405 |
| 77 | G/A |  |  | 0.45 | 0.435 | 0.33 | 1.00 | 0.580 | 0.495 | 0.478 | 0.491 |
| 78 | G/C |  |  | 0.346 | 0.456 | 0.4 | 0.96 | 0.872 | 0.492*** | 0.822 | 0.496*** |
| 79 | T/C | beta-tubulin | NS | 0.333 | 0.397 | 0.31 | 0.84 | 0.476 | 0.444 | 0.641 | 0.479* |
| 80 | G/A |  |  | 0.298 | 0.122 | 0.43 | 0.96 | 0.596 | 0.418** | 0.244 | 0.215 |
| 81 | T/C |  |  | 0.439 | 0.391 | 0.39 | 0.99 | 0.551 | 0.493 | 0.478 | 0.476 |
| 82 | C/G |  |  | 0.01 | 0.337 | 0.47 | 0.99 | 0.020 | 0.020 | 0.457 | 0.447 |
| 83 | C/G |  |  | 0 | 0.109 | 0.4 | 1.00 | - | - | 0.130 | 0.194* |
| 84 | G/A |  |  | 0 | 0.174 | 0.35 | 0.99 | - | - | 0.304 | 0.287 |
